# Microbial Biosynthesis of L-Malic Acid and Related Metabolic Engineering Strategies: Advances and Prospects

**DOI:** 10.3389/fbioe.2021.765685

**Published:** 2021-09-29

**Authors:** Zhen Wei, Yongxue Xu, Qing Xu, Wei Cao, He Huang, Hao Liu

**Affiliations:** ^1^ MOE Key Laboratory of Industrial Fermentation Microbiology, College of Biotechnology, Tianjin University of Science & Technology, Tianjin, China; ^2^ School of Food Science and Pharmaceutical Engineering, Nanjing Normal University, Nanjing, China; ^3^ Tianjin Engineering Research Center of Microbial Metabolism and Fermentation Process Control, Tianjin University of Science & Technology, Tianjin, China

**Keywords:** L-malic acid, biosynthesis, Metabolic Engineering, fermentation, by-products

## Abstract

Malic acid, a four-carbon dicarboxylic acid, is widely used in the food, chemical and medical industries. As an intermediate of the TCA cycle, malic acid is one of the most promising building block chemicals that can be produced from renewable sources. To date, chemical synthesis or enzymatic conversion of petrochemical feedstocks are still the dominant mode for malic acid production. However, with increasing concerns surrounding environmental issues in recent years, microbial fermentation for the production of L-malic acid was extensively explored as an eco-friendly production process. The rapid development of genetic engineering has resulted in some promising strains suitable for large-scale bio-based production of malic acid. This review offers a comprehensive overview of the most recent developments, including a spectrum of wild-type, mutant, laboratory-evolved and metabolically engineered microorganisms for malic acid production. The technological progress in the fermentative production of malic acid is presented. Metabolic engineering strategies for malic acid production in various microorganisms are particularly reviewed. Biosynthetic pathways, transport of malic acid, elimination of byproducts and enhancement of metabolic fluxes are discussed and compared as strategies for improving malic acid production, thus providing insights into the current state of malic acid production, as well as further research directions for more efficient and economical microbial malic acid production.

## Introduction

L-malic acid is a ubiquitous dicarboxylic acid found in all organisms, but its name derives from the fact that it was first isolated from unripe apples in 1785 (Meek, 1975). In 1967, it was classified as a safe food-grade product by the U.S. Food and Drug Administration (FDA). Currently, malic acid is mainly used as an acidulant and flavor enhancer in the food and beverage industries. Due to its more intense acid taste and better taste retention compared with citric acid, L-malic acid is becoming one of the most widely used organic acidulants. In the pharmaceutical industry, L-malic acid is used to improve the absorption of drugs and is used in amino acid infusions for the treatment of liver dysfunction or high blood ammonia (Chi et al., 2014). A mixture of calcium citrate and calcium malate is a commonly used source of calcium for improved bone strength without increasing the risk of kidney stones (Thakker et al., 2015). Other commercial applications include metal cleaning, finishing, animal feed and chemical synthesis of biodegradable polymers, such as polymalic acid (PMA) (Goldberg et al., 2006; Dai et al., 2018). Malic acid was listed as one of the top twelve bio-based building block chemicals by the US Department of Energy (Werpy and Petersen, 2004). The current global malic acid production capacity is estimated between 80,000 and 100,000 tons per year, while the annual market demand is estimated at over 200,000 tons, with a steadily rising market potential (Sauer et al., 2008; Zou et al., 2015).

The primary commercial production of malic acid is currently based on petrochemical routes, such as the hydration of maleic anhydride generated from the oxidation of benzene or butane at high temperature and high pressure, yielding a racemic mixture of D- and L-malic acid (Naude and Nicol, 2018). Malic acid has an asymmetric carbon and therefore it occurs in two isomers. Enantiopure L-malic acid is the physiological form present in all living organisms, ranging from bacteria to humans, while D-malic acid is rare in nature and difficult to assimilate by humans, thus it is not applicable to very young infants and elderly people. In 1970, the U.S. FDA ruled that DL-malic acid could not be used as an additive in infant food. Enzymatic conversion is an alternative process for synthesis of L-malic acid, using either immobilized fumarate hydratase or whole cells (*Brevibacterium ammoniagenes* or *Saccharomyces cerevisiae*) containing the enzyme fumarate hydratase to catalyze the conversion of fumarate into malic acid (Chibata et al., 1987; Peleg et al., 1988; Knuf et al., 2014). However, the expensive purification of fumarate hydratase and difficult separation of L-malic acid from the unreacted substrate greatly increased the cost of L-malic acid production. In addition, substrates such as maleic anhydride or fumarate are derived from non-sustainable petroleum, and the upward trend in the cost of finite petroleum resources further hampered the expansion of the malic acid market (Goldberg et al., 2006; Liu et al., 2017a). With the increasingly severe challenges related to the depletion of fossil-based resources as well as environmental issues, ecofriendly sustainable microbial fermentative production of malic acid has been given more attention. A lot of progresses has been made in the development of engineered strains or processes in recent years.

The focus of this review concerns the latest progresses on malic acid production, biosynthetic pathways and metabolic engineering strategies. By summarizing the major progress in metabolic engineering strategies in various microbes, encompassing the enhancement of biosynthetic pathways, transportation systems and metabolic fluxes, as well as eliminating by-product pathway for improving malic acid production, this review aims to provide a valuable reference for future development of microbes as cell factories for industrial production of malic acid.

## Malic Acid Production Using Wild Type Microorganisms

As an intermediate of the TCA cycle, malic acid can be accumulated as a fermentation end-product by various microorganisms including filamentous fungi, yeasts, and bacteria. However, only a few wild-type filamentous fungi such as *Aspergillus* (Battat et al., 1991) and *Penicillium* (Wang et al., 2013; Khan et al., 2014) have the native ability to produce malic acid in large quantities from glucose and other carbon sources (Table 1).

**TABLE 1:** Malic acid production by wild type microorganisms.

Microorganisms	Substrates	Titer (g/L)	Yield^a^ (mol/mol)	Productivity (g/L/h)	References
*Aspergillu flavus* ATCC 13697	Glucose	58^b^	0.84	0.1	Abe et al. (1962)
*Aspergillu flavus* ATCC 13697	Glucose	113^c^	1.26	0.59	Battat et al. (1991)
*Aspergillu niger* ATCC9142	Thin stillage	17^b^	0.8 (g/g)	0.09	West (2011)
*Aspergillus niger* ATCC10577	Thin stillage	19^b^	-	0.10	West (2011)
*Aspergillus niger* ATCC 12486	Crude glycerol	23^b^	-	0.12	West (2015)
*Aspergillus oryzae* NRRL 3488	Glucose	30.27^c^	0.98	0.89	Knuf et al. (2013)
*Aspergillus oryzae* DSM1863	Glucose	58.2^b^	0.76	0.16	Ochsenreither et al. (2014)
*Aspergillus oryzae* DSM1863	Glycerol	45.43^b^	0.37 (g/g)	0.13	Ochsenreither et al. (2014)
*Aspergillus oryzae* DSM1863	Xylose	39.40^b^	0.49 (g/g)	0.11	Ochsenreither et al. (2014)
*Penicillium viticola* 152	Glucose	131^c^	1.34	1.36	Khan et al. (2014)
*Penicillium sclerotiorum* K302	Glucose	71.67^c^	0.93	1.00	Wang et al. (2013)
*Saccharomyces cerevisiae*	Glucose	1^b^	-	-	Fatichenti et al. (1984)
*Saccharomyces cerevisiae*	Glucose	2^b^	-	-	Schwartz and Radler (1988)
*Schizophyllum commune*	Glucose	18^c^	0.48	0.18	Kawagoe et al. (1997)
IFO-4928					
*Zygosaccharomyces rouxii* V19	Glucose	74.90^d^	0.52	0.21	Taing and Taing (2007)

a Yields are given in mol malic acid per mol glucose unless otherwise indicated.

b Flask culture.

c Fermentor culture.

d Test tube - represents no value.

*Aspergillus* species are well known strains for malic acid production. *Aspergillus flavus* was the first patented strain for malic acid production in 1963 (Abe et al., 1962). Through process optimization, *A. flavus* achieved a maximal malic acid titer of 113 g/L in 190 h (Battat et al., 1991). However, it has never been applied for large-scale production of malic acid due to its production of carcinogenic aflatoxins during the fermentation process (Battat et al., 1991; Geiser et al., 1998). *Aspergillus oryzae* is generally regarded as safe (GRAS). Given its high similarity with *A. flavus*, *A. oryzae* NRRL 3488 was investigated for the production of malic acid. A titer of 30.27 g/L malic acid was obtained with a yield of 0.98 mol/mol under high glucose and nitrogen starvation conditions (Knuf et al., 2013). *A. oryzae* DSM1863 was used to produce malic acid from the waste substrate glycerol and the renewable carbon source xylose as, reaching product titers of 39.40 and 45.43 g/L, respectively (Ochsenreither et al., 2014). *Aspergillus niger* is a well-known industrial workhorse for the production of organic acids, and its application for malic acid production has received increasing attention in recent years. *A. niger* strains ATCC 9142 and ATCC 10577 were investigated for the production of malic acid from thin stillage, and achieved product titers of 17 and 19 g/L, respectively (West, 2011). When using crude glycerol as feedstock, *A. niger* ATCC 12486 could produce 23 g/L malic acid after 192 h at 25°C (West, 2015).

Several *Penicillium* species such as *P. viticola* 152 and *P. sclerotiorum* K302 isolated from marine environments were reported to be good malic acid producers, respectively accumulating up to 131 and 71.67 g/L L-malic acid from glucose in 10 L fermenters. The titer of 131 g/L with a yield of 1.34 mol/mol glucose and a productivity of 1.36 g/L/h represents the highest malic acid production achieved using *Penicillium* to date (Wang et al., 2013; Khan et al., 2014).

Yeasts such as *S*. *cerevisiae* are excellent platforms for the biological production of industrial chemicals and have also been investigated for fermentative malic acid production. Malic acid was detected as a by-product in the yeast fermentation process as early as 1924 (Yin et al., 2015). Afterwards, at least eight *S. cerevisiae* strains were tested in flasks to produce malic acid, but only two strains could synthesize more than 1 g/L of malic acid in 7 days (Fatichenti et al., 1984). *Zygosaccharomyces rouxii* is an osmotolerant yeast associated with foods of low water activity. *Z. rouxii* V19 isolated from high-sugar fermented foods was able to produce 74.90 g/L of malic acid with a yield of 0.52 mol/mol from 193 g/L glucose within 15 days under optimized conditions (Taing and Taing, 2007).

Some mushrooms also produce useful materials such as organic acids, and *Schizophyllum commune* IFO-4928 was able to produce 18 g/L of malic acid from glucose under optimized conditions (Kawagoe et al., 1997). In addition, the yeast-like fungus *Aureobasidium pullulans* was identified as a proficient polymalic acid (PMA) producer, reaching a product titer of 47 g/L from glucose (Nagata et al., 1993). PMA is a linear anionic C_4_-polyester consisting of L-malic acid monomers. Recently, many researchers attempted to produce malic acid through the hydrolysis of PMA (Zou et al., 2013; Zou et al., 2015), and 144.2 g/L L-malic acid was obtained following purification and hydrolysis of 123.7 g/L PMA, which was produced by fed-batch fermentation using cells immobilized in a fibrous-bed bioreactor (FBB). This process provided a novel approach for malic acid production (Zou et al., 2013).

## Malic Acid Production by Mutants and Evolutionarily Engineered Microorganisms

Conventional mutagenesis based on soft X-rays, atmospheric and room temperature plasma (ARTP) or mutagenic chemicals was used to generate highly productive strains of *Rhizopus delemar*, *A. oryzae* and *Monascus araneosus* (Table 2). In addition, adaptive laboratory evolution was frequently used to improve microbial characteristics such as the ability to utilize non-preferred carbon sources for malic acid production (Zambanini et al., 2016b; Iyyappan et al., 2018b).

**TABLE 2:** Malic acid production by mutants and evolutionarily engineered microorganisms.

Microorganisms	Substrates	Titer (g/L)	Yield (g/g)	Productivity (g/L/h)	References
*Aspergillus niger* MTCC 281	Crude glycerol	77.38^a^	-	0.40	Iyyappan et al. (2018b)
*Aspergillus niger* PJR1	Crude glycerol	83.23^a^	-	0.43	Iyyappan et al. (2018a)
*Aspergillus niger* PJR1	Crude glycerol	92.64^a^	-	0.48	Iyyappan et al. (2019b)
*Aspergillus oryzae* FMME218-37	Glucose	95.2^b^	0.54	0.57	Ding et al. (2018)
*Monascus araneosus*	Glucose	27.9^a^	0.37	0.23	Lumyong and Tomita (1993)
*Rhizopus delemar*	Corn straw	120^b^	0.96	2.03	Li et al. (2014)
*Ustilago trichophora* TZ1	Crude glycerol	195^b^	0.43	0.74	Zambanini et al. (2016a)
*Ustilago trichophora* TZ1	Crude glycerol	196^a^	0.82	0.39	Zambanini et al. (2016b)

a Flask culture.

b Fermentor culture - represents no value.

Using random mutagenesis and screening processes, a mutant of the zygomycete fungus *R*. *delemar* HF-121 was obtained that could produce more than 120 g/L malic acid from corn straw hydrolysate in a pilot-scale fermenter within 60 h. Moreover, it exhibited the highest malic acid productivity reported to date, reaching 2.03 g/L/h. The high malic acid production from biomass hydrolysate highlights the prospect of large-scale industrial application of this strain (Li et al., 2014). *A. oryzae* is an efficient malic acid-producing strain, and organic nitrogen is more favorable to the production of malic acid than inorganic nitrogen added in the fermentation culture. To meet the requirements of a low-cost nitrogen sources, Ding et al. (2018) constructed a library of mutants with (NH_4_)_2_SO_4_ as the sole nitrogen source for L-malate production. Briefly, *A. oryzae* spores were firstly treated with atmospheric and room temperature plasma (ARTP) and ten mutants with relatively higher L-malate titers were selected out in the mutant library. The analysis of these 10 mutants revealed that the production of L-malate was positively related with the colony diameter (*D*) and the specific surface area of unit volume (S2). Furtherly, the mutant with the highest L-malate production was treated with ^60^Co-γ radials and DES solution. Using the above two parameters as the basis for selection, three strains with significantly increased L-malate production were finally obtained. The final titer and productivity of malic acid reached 95.2 g/L and 0.57 g/L/h in a 7.5 L fermenter, which represents the highest level achieved to date in *A. oryzae* using an inorganic nitrogen source (Ding et al., 2018). In addition, an albino mutant was isolated following N-methyl-N′-nitro-N-nitrosoguanidine treatment of *Monascus araneosus* AHU9087, which was able to produce 27.9 g/L malic acid after 5 days at 37°C under aerobic conditions, compared with 20 g/L produced by the parent strain (Lumyong and Tomita, 1993).

Geiser et al. (2014) screened 68 members of the family Ustilaginaceae for the production of organic acids, and found that many strains can accumulate organic acids, including malic acid (Geiser et al., 2014). *Ustilago trichophora* TZ1 was found to efficiently produce malic acid from glycerol. Following adaptive laboratory evolution and process optimization, the final malic acid titer, yield and overall productivity respectively reached 196 g/L, 0.82 g/g and 0.39 g/L/h in shake flasks (Zambanini et al., 2016b), as well as 195 g/L, 0.43 g/g, 0.74 g/L/h in a fed-batch bioreactor within 264 h (Zambanini et al., 2016a). However, the potential plant pathogenicity and limited genetic information are the major drawbacks of using *Ustilago* spp. in large-scale processes. Considering the important industrial value of *A. niger*, the mutant strain MTCC 281 with dual resistance to methanol and malic acid was obtained using an adaptation process spanning 22 weeks. The yield of malic acid from crude glycerol increased 4.45-fold compared with that of the parent strain, and the highest product titer reached 77.38 g/L after 192 h at 25°C (Iyyappan et al., 2018b). In a different approach, the malic acid titer was increased to 83.23 g/L by using morphologically controlled *A. niger* in the form of dispersed fungal mycelium in shake flask culture (Iyyappan et al., 2018a). After further process optimization, the maximal titer of malic acid reached up to 96.24 g/L (Iyyappan et al., 2019b).

## Malic Acid Production by Genetically Engineered Microorganisms Using Metabolic Engineering Strategies

**FIGURE 1:**
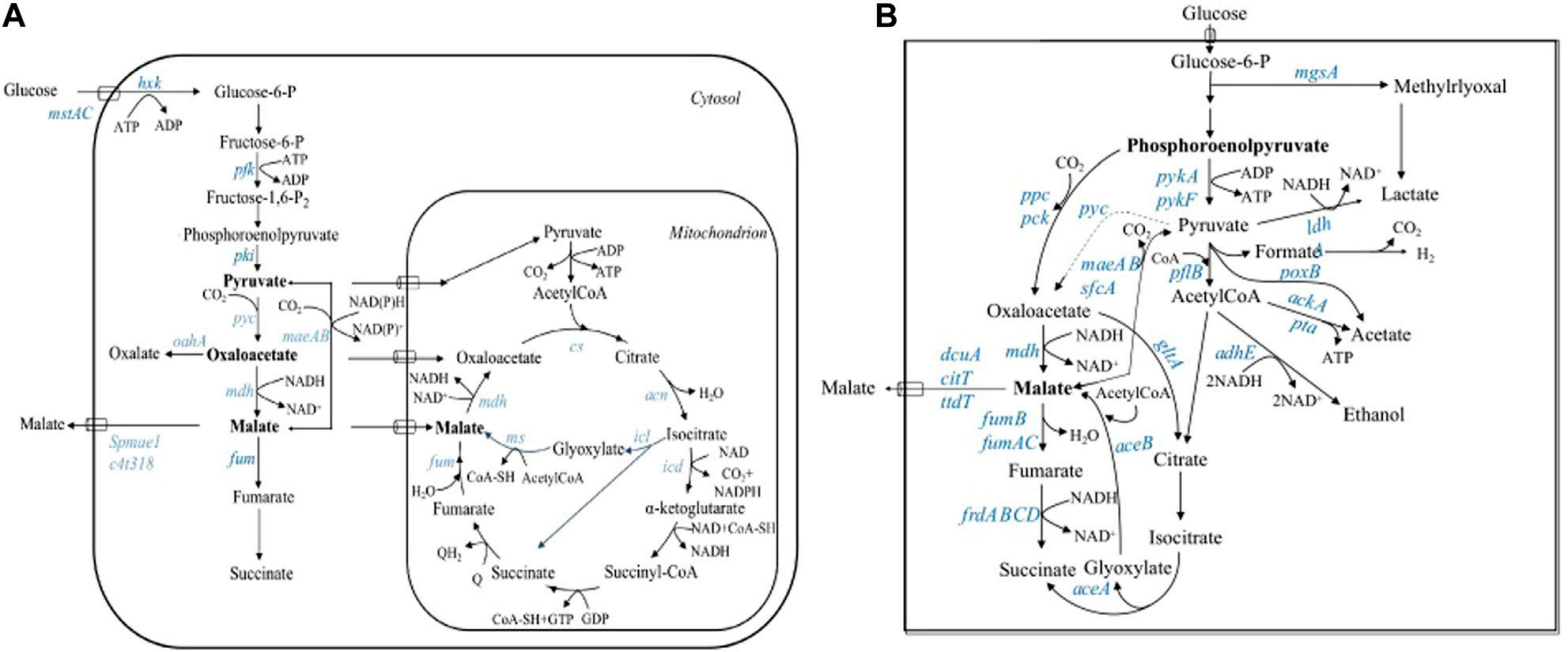
Biosynthetic pathways of L-malic acid in eukaryotes **(A)** and prokaryotes **(B)**. The dotted line indicates an exogenous pathway that does not exist in the natural strain. Enzyme-coding genes that were manipulated through genetic engineering are depicted in blue. Abbreviation: Panel **(A)**: *acn*, aconitase; *cs*, citrate synthase; *c4t318*, malate transporter from *Aspergillus oryzae*; *frd*, fumarate reductase; *fum*, fumarase; *hxk*, hexokinase; *icd*, isocitrate dehydrogenase; *icl*, isocitrate lyase; *maeAB*, malic enzyme; *mdh*, malate dehydrogenase; *ms*, malate synthetase; *mstA/C*, monosaccharide transporter; *oahA*, oxaloacetate acetylhydrolase; *pfk*, phosphofructokinase; *pki*, pyruvate kinase; *pyc*, pyruvate carboxylase; *Spmae1*, malate transporter from *Schizosaccharomyces pombe.* Panel **(B)**: *aceA*, isocitrate lyase; *aceB*, malate synthase; *ackA*, acetate kinase; *adhE*, alcohol dehydrogenase; *citT*, citrate transporter; *dcuA*, dicarboxylate uptake transporter; *frdABCD*, fumarate reductase; *fumB/fumAC*, fumarase; *gltA*, citrate synthase; *ldhA*, D-lactate dehydrogenase; *mgsA*, methylglyoxal synthase; *pck*, phosphoenolpyruvate carboxykinase; *pflb*, pyruvate formate-lyase; *poxB*, pyruvate oxidase; *ppc*, phosphoenolpyruvate carboxylase; *pta*, phosphate acetyltransferase; *pyk*, pyruvate kinase; *sfc*, succinate/fumarate transporter; *ttdT*, tartrate *transporter.*

As stated above, various wild-type and laboratory-evolved microorganisms have been investigated for fermentative production of malic acid, but the product yield or productivity is usually low and far from the requirements of large-scale industrial production (Table 1). With the increasing development of metabolic engineering and synthetic biology in the past decade, the engineering of biosynthetic pathways has become a viable approach for the construction of efficient microbial cell factories (Zhou et al., 2012; Choi et al., 2016; Chen et al., 2020). In recent years, many efficient L-malic acid production strains have been developed by redesign of biosynthetic pathways or transport systems and blocking the formation of byproducts (Table 3). Several metabolic pathways have been used to synthesize L-malic acid, including the reductive TCA (rTCA) pathway, the conversion of phosphoenolpyruvate into oxaloacetate, the tricarboxylic acid (TCA) cycle, the glyoxylate pathway, and direct one-step conversion of pyruvate into malic acid (Figure 1).

**TABLE 3:** Malic acid production by metabolically engineered microorganisms.

Microorganisms	Titer (g/L)	Yield^a^ (mol/mol)	Productivity (g/L/h)	Main by-products (g/L)	Genetic modifications	References
*Aspergillus niger* S1149	201.13^b^	1.64	1.05	Fumarate (1.50–1.80)	Δo*ahA*, Δ*cexA*, OE*pyc*, OE*mdh3*, OE *c4t318*, OE*mstC*, OE*hxkA*, OE*pfkA*, OE*pkiA*	Xu et al. (2020)
*Aspergillus niger* S575	201.24^b^	1.27	0.93	Citrate (28.00)	Δo*ahA*, OE*pyc*, OE*mdh3* and OE *c4t318*	Xu et al. (2019)
*Aspergillus oryzae* 2103a-68	154^b^	1.38	0.94	Succinate (13) and citrate (6)	OE*pyc*, OE*mdh*3 and OE*c4t318*	Brown et al. (2013)
*Aspergillus oryzae*	165^b^	0.91	1.38	Succinate (18.7) and fumarate (4.0)	OE*pyc*, OE*mdh*, OE*Ecppc*, OE*Ecpck*, OE*c4t318*, OE*SpMAE*1 and OE*pfk*	Liu et al. (2017a)
*Aspergillus oryzae*	82.3^c^	0.82 g/g (corn starch)	1.18	-	OE*glaA*, OE*amyB*, OE*agdA* and OE*Scfum1*	Liu et al. (2017b)
*Aspergillus oryzae*	117.2^c^	0.9 g/g (corn starch)	1.17	Succinate (3.8) and fumarate (0.75)	OE*Ropyc*, OE*icl1*, OE*icl2*, OE*mas*, DR*cs*, OE*Sfc1p* and OE*nox*	Liu et al. (2018)
*Aspergillus carbonarius*	32.0^c^	-	0.15	Succinate (16.0) and citrate (5.2)	OE*dct*	Yang et al. (2017b)
*Bacillus subtilis*	2.1^c^	0.16	0.03	Acetate (3.14)	Δ*ldh*, OE*Ecppc* and OE*Scmdh*	Mu and Wen (2013)
*Escherichia coli* KJ071	69.1^b^	1.4	0.48	Succinate (33.07) and pyruvate (5.1)	Δ*ldhA*, Δ*adhE*, Δ*ackA*, Δ*focA*, Δ*pfB* and Δ*mgsA*	Jantama et al. (2008)
*Escherichia coli* XZ658	34^b^	1.42	0.47	Succinate (1.18) and lactate (1.08)	Δ*ldhA*, Δ*ackA*, Δ*adhE*, Δ*pflB*, Δ*mgsA*, Δ*poxB*, Δ*frdBC*, Δ*sfcA*, Δ*maeB*, Δ*fumB* and Δ*fumAC*	Zhang et al. (2011)
*Escherichia coli*	36.05^b^	0.74	0.60	-	OE*Afpyc*, OE*Scms*, OE*cs*, OE*acn* and OE*icl*	Gao et al. (2018)
*Escherichia coli* F0931	21.65^b^	0.48	0.3	Pyruvate (16.54) and succinate (0.98)	Δ*ldhA*, Δ*poxB*, Δ*pflB*, Δ*pta*, Δ*ackA*, Δ*frdBC*, Δ*fumABC*, OE*me* and OE*pos5*	Dong et al. (2017)
*Escherichia coli*	-	0.82	-	fumarate (-)	Δ*mdh*, Δ*mqo*, Δ*maeAB,* Δ*iclR arcA*, OE*ppc* and OE*gltA*	Trichez et al. (2018)
*Escherichia coli* GL2306	25.86^c^	0.53	0.36	-	Δ*adhE*, Δ*ackA*, Δ*ldhA*, Δ*pts1*, Δ*pflB*, Δ*focA*, Δ*mgsA*, OE*Ecpck* and OE*Asmdh*	Guo et al. (2018)
*Escherichia coli*	17.83^b^	1.3	0.38	-	Δ*ldhA*, Δ*adhE*, Δ*iclR*, Δ*ack*, Δ*pta* and OE*pyc*	Martinez et al. (2018)
*Escherichia coli* MA-11	5.90^c^	0.80 g/g (xylose)	0.08	Glycolate (-)	Δ*maeA*, Δ*maeB*, Δ*mdh*, Δ*fumAC*, Δ*fumB*, OE*dte*, OE*fucA*, OE*fucK*, OE*aldA*, OE*glcDEFB* and OE*katE*	Li et al. (2018)
*Escherichia coli* XL-1	12.08^c^	-	-	-	OE*PykF* and OE*SfcA*	Somasundaram et al. (2018)
*Myceliophthora thermophile* J207	181^b^	- (avicel)	-	Succinate (19.7)	OE*Aoc4t318 and* OE*Aopyc*	Li et al. (2019)
*Pichia pastoris*	42.28^b^	0.56	0.44	Succinate (9.42)	OE*pyc* and OE*mdh1*	Zhang et al. (2015)
*Saccharomyces cerevisiae* MDH	11.8^c^	0.13	0.38	Citrate (40.7)	OE*mdh2*	Pines et al. (1997)
*Saccharomyces cerevisiae* RWB525	59^c^	0.42	0.19	succinate (8.0) and glycerol (25.0)	OE*pyc2*,OE*mdh3*Δ*SKL* and OE*Spmae1*	Zelle et al. (2008)
*Saccharomyces cerevisiae* RWB525	35.91^b^	0.48	-	Pyruvate (30.54) and succinate (11.33)	OE*pyc2*,OE*mdh3*Δ*SKL* and OE*Spmae1*	Zelle et al. (2010)
*Saccharomyces cerevisiae*	30.25^c^	0.4	0.32	Pyruvate (30.75)	OE*Afpyc*, OE*Ropyc*, OE*Afmdh*, OE*Romdh* and OE*SpMAE1*	Chen et al. (2017)
*Torulopsis glabrata*	8.5^c^	0.19	0.18	Pyruvate (-)	OE*Ropyc*, OE*Romdh* and OE*SpMAE1*	Chen et al. (2013)
*Thermobifida fusca muC-16*	62.76^b^	0.63 g/g (cellulose)	0.51	Succinate (2.40) and bytyrate (11.1)	OE*C*g*pyc*	Deng et al. (2016)
*Ustilago trichophora* TZ1	134^b^	0.42 g/g (glycerol)	0.56	Succinate (20) and α-ketoglutarate (8)	OE*pyc*, OE*mdh1*, OE*mdh2*, OE*ssu1* and OE*ssu2*	Zambanini et al. (2017)

a Yields are given in mol malate per mol glucose unless otherwise indicated.

b Fermentor culture.

c Flask culture - represents no value.

OE, overexpression; DR, down-regulation.

### Combined Enhancement of the rTCA Pathway and Malic Acid Transport

The role of the rTCA pathway in L-malic acid production was first demonstrated in *A. flavus* using NMR-based metabolic flux analysis with 1-^13^C-laballed glucose as carbon source (Peleg et al., 1988). This pioneering study paved the way for targeted metabolic engineering towards efficient L-malic acid production. Subsequently, this pathway was also identified in *A. niger*, *S*. *cerevisiae* and *A. oryzae* using similar ^13^C NMR experiments (Peksel et al., 2002; Zelle et al., 2008; Knuf et al., 2014). These analyses clearly showed that rTCA is the predominant pathway for extracellular malic acid accumulation. The rTCA pathway takes place in the cytosol and involves the carboxylation of pyruvate to oxaloacetate, followed by the reduction of oxaloacetate to malic acid (Figure 1A). Pyruvate carboxylase (Pyc) is the first key enzyme in the rTCA pathway, catalyzing the ATP-dependent condensation of pyruvate and CO_2_ to form oxaloacetate (Goldberg et al., 2006; Dai et al., 2018). Generally, Pyc is situated in mitochondria of eukaryotic cells. However, the enzyme is localized exclusively in the cytosol in certain filamentous fungi and *S*. *cerevisiae* due to the lack of a mitochondrial-targeting peptide (van Urk et al., 1989; Bercovitz et al., 1990; Goldberg et al., 2006; Khan et al., 2017). Malic acid dehydrogenase (Mdh) is the second enzyme that catalyzes the NAD(H)-dependent reversible conversion of malic acid into oxaloacetate. There are two forms of Mdh in eukaryotes, one of which is localized to the mitochondria and participates in the TCA cycle, while the other is localized to the cytoplasm and participates in the rTCA pathway (Goldberg et al., 2006). Additionally, a special form of malic acid dehydrogenase (Mdh3) found in *S. cerevisiae* was found to be localized in peroxisomes (Steffan and McAlister-Henn, 1992). During the acid production stage, the activity of malic acid dehydrogenase in *A. flavus* was found to be increased 6- to 10-fold compared with the growth stage, suggesting that Mdh is important for L-malic acid accumulation (Peleg et al., 1988; Battat et al., 1991). However, it was not clear which form of Mdh plays a major role in this process. Pyruvate is an important precursor for malic acid synthesis. If pyruvate is completely derived from the glycolytic pathway, then the ATP and redox reactions for malate synthesis *via* the rTCA pathway are balanced. In addition, 1 mol of CO_2_ is fixed in the carboxylation of 1 mol of pyruvate, which results in the maximal theoretical yield of malic acid being 2 mol/mol of glucose (Zelle et al., 2008). Given its high theoretical yield and relative simplicity, the rTCA pathway has been extensively re-designed to improve malic acid production in yeasts and filamentous fungi (Table 3).

Several yeasts can accumulate L-malic acid through the rTCA pathway (Pines et al., 1996), and efforts have been made to improve malic acid production in *S. cerevisiae* (Zelle et al., 2008; Chen et al., 2017). The impact of individual and combined introduction of the native Pyc2, Mdh3Δ*SKL* (deletion of the C-terminal peroxisomal targeting sequence) and malic acid transporter SpMae1 from *Schizosaccharomyces pombe* were evaluated in a *S. cerevisiae* TAM strain (van Maris et al., 2004; Zelle et al., 2008). Overexpression of *pyc2* alone and in combination with either *mdh3*Δ*SKL* or Sp*mae1* did not result in an increase of malic acid production, indicating that Pyc has a low degree of control over the rate of malic acid accumulation. By contrast, when malic acid dehydrogenase and a malic acid exporter were co-overexpressed, the control of malic acid production shifted towards Pyc. The highest malic acid yield (0.42 mol/mol) and titer (59 g/L) were obtained with the simultaneous introduction of all three modifications (Zelle et al., 2008). Under optimized conditions, the malic acid titer reached 35.91 g/L, with a yield of 0.48 mol/mol in bioreactor cultures (Zelle et al., 2010). Chen et al. (2017) reconstructed the rTCA pathway in *S. cerevisiae* TAM for malic acid biosynthesis by combinatorial overexpression of Af*pyc* (from *A. flavus*), Ro*mdh* (from *R. oryzae*) and Spmae* (encoding a mutated Mae transporter resistant to ubiquitination) at different expression levels. The maximal titer of malic acid reached 30.25 g/L during batch fermentation, which was lower than the previously reported 35.91 g/L (Chen et al., 2017). In addition, Ro*pyc*, Ro*mdh* and Sp*mae1* were heterologously overexpressed in *Torulopsis glabrata* to manipulate the carbon flux from pyruvate to malic acid, but the final titer of malic acid was only 8.5 g/L (Chen et al., 2013). *Pichia pastoris* was engineered by overexpressing the native *pyc* and *mdh1* genes, which resulted in a malic acid titer of 42.28 g/L after 96 h (Zhang et al., 2015).

*U. trichophora* was engineered to accumulate high levels of malic acid by adaptive laboratory evolutionary as mentioned above. In order to further improve the yield of malic acid on glycerol, two malic acid dehydrogenases (*mdh1*, *mdh2*), and two malic acid transporters (*ssu1*, *ssu2*) were overexpressed in *U. trichophora* TZ1 (Zambanini et al., 2017), which increased the malic acid yield by up to 54%, resulting in a titer of up to 120 g/L in shake flasks. However, the final titer in bioreactor cultivation was dramatically decreased to 134 g/L, which was much lower than earlier reports (Zambanini et al., 2016a; Zambanini et al., 2016b). In addition, overexpression of *pyc* alone resulted in lower or similar malic acid production compared to the reference strain (Zambanini et al., 2017).

In recent years, several attempts have been made to improve malic acid production by *A. oryzae via* genetic engineering. Overexpression of the endogenous transporter C4t318 resulted in a greater than two-fold increase of L-malic acid productivity, while the additional overexpression of *pyc* and *mdh*3 in the rTCA pathway led to a further increase by about 27%. The resulting strain overexpressing all three genes produced 154 g/L malic acid with a yield of 1.38 mol/mol and a productivity of 0.94 g/L/h (Brown et al., 2013). To further increase the production of malic acid, an oxaloacetate anaplerotic reaction was introduced to increase the supply of the oxaloacetate precursor by heterologous expression of the *ppc* (encoding phosphoenolpyruvate carboxylase) and *pck* (phosphoenolpyruvate carboxykinase) genes from *Escherichia. coli*, which improved the malic acid titer by 38.3% (Liu et al., 2017a). After further overexpression of the endogenous 6-phosphofructokinase (*pfk*) to strengthen the metabolic flux from glucose, the malic acid titer was increased to 165 g/L, with a yield and productivity of 0.68 g/g and 1.38 g/L/h, respectively, which represents the highest levels of malic acid production in *A. oryzae* reported to date (Liu et al., 2017a).

*Myceliophthora thermophila* can efficiently utilize cellulose and hemicellulose, which makes it a promising candidate for the production of C_4_-dicarboxylic acids. Both the *c4t318* and *pyc* genes from *A. oryzae* were heterologously overexpressed in *M. thermophila*, and the resulting strain JG207 was able to produce 65.4 g/L malic acid from 75 g/L Avicel^®^ in shake flask cultures (Li et al., 2019). With Avicel^®^ and corncob as the feedstock, 181 and 105 g/L of malic acid were produced in fed-batch fermentation, respectively. Transcriptional profiling analysis indicated that the cytosolic rTCA pathway was the main synthetic route of malic acid in strain JG207 grown on cellulose. After *ppc* gene form *E. coli* and the native cytoplasmic *mdh* were co-overexpressed, the malic acid titer increased to 72.4 g/L, with a yield of 0.96 g/g, which was higher than that of the parental strain (0.88 g/g). To increase the supply of CO_2_ in the cytoplasm, a CO_2_ concentration pathway was constructed by integrating *bicA* (HCO_3_^−^ transporter gene) and *ca* (carbonic anhydrase gene) from *Synechococcus* sp. PCC7002 into the loci of *pdc* (encoding pyruvate decarboxylase) and *ldh* (acetate dehydrogenase), respectively, thereby reducing competing pyruvate consumption at the same time. The resulting strain displayed a further 15% increase of malic acid production, with a titer of 83.3 g/L and a yield of 1.11 g/g in shake flasks with Avicel^®^ as the substrate (Li et al., 2019).

Most recently, *A*. *niger* has been successfully engineered for fermentative production of malic acid by deleting the oxaloacetate acetylhydrolase gene (*oahA*) and overexpressing the endogenous rTCA pathway genes *pyc* and *mdh3*, combined with the insertion of the malic acid transporter gene *c4t318* from *A. oryzae*. The malic acid titer of the engineered strain S575 was increased over 5-fold compared with the wild-type strain, reaching 120.38 g/L in shake-flask culture. Moreover, this titer was increased to 201.24 g/L in fed-batch fermentation, with an overall productivity of 0.93 g/L/h, and yield of 1.27 mol/mol glucose (Xu et al., 2019). In *A. niger*, oxaloacetate is the common precursor of oxalic acid and malic acid biosynthesis through the rTCA pathway (Figure 1A). Deletion of *oahA* blocked the oxalic acid synthesis, resulting in a 40% increase of the malic acid titer. Further enhancement of the rTCA pathway and overexpression of the malic acid transporter C4t318 led to an additional 4-fold increase of the product titer. The resulting titer of 201.24 g/L is the highest level of malic acid production reported to date, and is close to the requirements of industrial production (Xu et al., 2019). Therefore, the strategy of combinational enhancement of the rTCA pathway and malic acid transport is the most commonly employed and remarkably effective was to improve malic acid production in yeasts and filamentous fungi. In addition, increasing the supply of precursors such as oxaloacetate and CO_2_ is also important for further improvement of malic acid production (Liu et al., 2017a; Li et al., 2019).

### Engineering the Non-Oxidative Pathway for Malic Acid Production

Different from filamentous fungi and yeasts, in which the rTCA pathway was engineered to improve malic acid production, boosting the conversion of phosphoenolpyruvate (PEP) into oxaloacetate by overexpressing *ppc* or *pck*, followed by reduction to malic acid, is a common strategy for the production of malic acid in *E. coli*, which has no pyruvate carboxylase (Li Q. et al., 2016) (Figure 1B). Zhang et al. (2011) confirmed that disruption of *pck* in *E. coli* XZ658 led to a dramatic decrease of malic acid production (Zhang et al., 2011). Although *E. coli* has been genetically modified for L-malate production, the wild-type didn’t exhibit a potential in L-malate secretion (Kövilein et al., 2020). Similarly, no malic acid was produced by the host *E. coli* WGS-10. By strengthening the supply of the precursor substance oxaloacetate by homologous or heterogenous overexpression of *pckA*, L-malate titers increased to 1.42 g/L and 9.25 g/L, respectively (Moon et al., 2008). More recently, Guo et al. (2018) constructed the dual regulation strain *E. coli* GL2306 by first deleting multiple genes (*adhE*, *ackA*, *ldhA*, *pts1*, *pflB*, *focA* and *mgsA*) to increase the PEP pool, and then co-overexpressing *Ecpck* and *Asmdh* from *Actinobacillus succinogenes* to reconstruct the malic acid biosynthesis pathway, which was targeted to the periplasm and cytoplasm using appropriate signal peptides. The final engineered strain GL2306 produced 25.86 g/L malic acid with a yield of 0.53 mol/mol in a 5-L two-stage fed-batch process (Guo et al., 2018). *B. subtilis* was also metabolically engineered to produce malic acid by heterologous overexpression of *ppc* (from *E. coli*) and *mdh2* (from *S. cerevisiae*) and deletion of *ldh*. The resulting strain produced a low titer of malic acid (2.01 g/L), but it represents the first engineered *B. subtilis* for malic acid production (Mu and Wen, 2013). Additionally, the impact of heterologous expression of Pyc for malic acid production has also been investigated in prokaryotes such as *E. coli* and *Thermobifida fusca*. Several metabolically engineered *E. coli* strains were constructed by single, double and triple deletion of *fumABC* in the background of a Δ*ldhA*/Δ*adhE* double mutant, together with the overexpression of *pyc* from *Lactococcus lactis*. Only the *fumAC* and the triple *fumABC* deletion strains accumulated malic acid as the main C4-dicarboxylic acid product with a yield of 0.61–0.67 mol/mol glucose (Martinez et al., 2018). Overexpression of an exogenous *pyc* gene from *Corynebacterium glutamicum* in *T. fusca* muC resulted in a 47.9% increase of the malic acid yield compared to the parental strain. The final strain *T. fusca* muC-16 was cultured on glucose and milled corn stover, which resulted in malic acid titers of 62.76 and 21.47 g/L, respectively. These studies were conducted in batch fermentation under low oxygen conditions, and butyrate was accumulated as the main by-product (Deng et al., 2016).

### Engineering the TCA Cycle for Malic Acid Production

Malic acid is an intrinsic intermediate of the TCA cycle, in which oxaloacetate and acetyl-coenzyme A (acetyl-CoA) are condensed to citrate followed by several oxidative reactions to form malic acid. Because two CO_2_ molecules are released during the oxidation of citrate to malic acid, the maximal theoretical yield is limited to 1 mol/mol glucose. Recently, Trichez et al. (2018) engineered *E. coli* for malic acid production *via* the TCA cycle, and found that achieving a significant yield of malic acid (0.82 mol/mol) required at least the simultaneous deletion of all malic enzymes and dehydrogenases (*Δmdh*, *Δmqo*, *ΔmaeAB*, *ΔiclR* and *ΔarcA*), with the concomitant expression of the malic acid-insensitive PEP carboxylase mutant Ppc^K620S^ and NADH-insensitive citrate synthase mutant GltA^R164L^. However, metabolic flux analysis based on ^13^C-labeled glucose indicated that the malic acid-producing strains had a very high flux through the glyoxylate shunt, with almost no flux passing through the isocitrate dehydrogenase (Icd) reaction. Generally, the TCA cycle oxidizes citrate into malic acid through a series of reactions under aerobic conditions, yet the highest malic acid production was achieved anaerobically (Trichez et al., 2018). In addition, although malic acid production through the TCA cycle can be achieved *in E. coli*, the fumarate by-product was also significantly accumulated, which makes this strategy unattractive compared to non-oxidative pathways (Trichez et al., 2018).

Liu et al. (2018) engineered the TCA cycle for malic acid production in *A. oryzae* by simultaneously overexpressing citrate synthase (CIS), isocitrate dehydrogenase (ISD), α-oxoglutarate dehydrogenase (OXD) and aconitate hydratase (ACH). However, the enhanced oxidative TCA cycle was unfavorable for malic acid synthesis, and actually decreased the malic acid titer from 95.1 to 83.7 g/L in corn starch culture. Conversely, appropriately downregulating the oxidative branch of the TCA cycle increased the carbon flux toward the rTCA pathway (Liu et al., 2018).

### Engineering the Glyoxylate Metabolism for Malic Acid Production

In bacteria and certain species of fungi, glyoxylate metabolism proceeds either *via* the glyoxylate cycle or the noncyclic glyoxylate shunt. Isocitrate lyase (Icl) and malic acid synthetase (Ms) are the two crucial enzymes in this pathway. Isocitrate lyase converts the isocitrate formed in the TCA cycle into succinate and glyoxylate, followed by the condensation of glyoxylate with acetyl CoA by malic acid synthetase (Iyyappan et al., 2019a). The glyoxylate shunt has not been found to be reversible to date (Mainguet et al., 2013). However, the synthesis of acetyl-CoA from pyruvate is accompanied by carbon loss due to CO_2_ release, which constrains the maximal theoretical malic acid yield in the glyoxylate cycle to 1 mol/mol glucose. If oxaloacetate is replenished by the carboxylation of pyruvate, the glyoxylate pathway is noncyclic, and the maximal malic acid yield increases to 1.33 mol/mol.

Recent studies have focused on the glyoxylate pathway for malic acid production. Although the overexpression of Icl in *A. niger* did not increase the production of malic acid (Meijer et al., 2009), the co-overexpression of Icl and Ms in *A. oryzae* increased the malic acid titer from 95.1 L to 99.8 g/L (Liu et al., 2018). To test the possibility of enhancing the malic acid yield of *E. coli* in aerobic fermentation based on the noncyclic glyoxylate shunt, Gao et al. (2018) recently established a five-enzyme (Pyc, Cs, Acn, Icl, Ms) cascade by integrating *in vitro* modular engineering with *in vivo* multiplexed CRISPRi tuning. The final strain *E. coli* B0013-47 exhibited a 2.3-fold higher malic acid titer than the parent, reaching 36 g/L with a yield of 0.74 mol/mol glucose in fed-batch fermentation. The advantage of this strategy is that the disbalance between different modules such as the accumulation of citrate and α-ketoglutarate can be solved using CRISPRi (Gao et al., 2018).

### Constructing a One-Step Pathway for Malic Acid Production

The one-step pathway involves the direct conversion of pyruvate into malic acid catalyzed by malic enzyme using NAD^+^/NADP^+^ as cofactor. Since there are no intermediates, carbon loss is minimized and the substrate transmission rate is improved in this pathway, which makes it possible to maximize the carbon flux for malic acid synthesis (Dong et al., 2017). The one-step conversion of pyruvate from glycolysis into malic acid *via* the malic enzyme has a theoretical yield of 2 mol/mol (Dong et al., 2017). However, malic enzyme catalyzes the reversible oxidative decarboxylation of malic acid into pyruvate and CO_2_, and the forward reaction (malic acid into pyruvate) is thermodynamically favorable due to the enzyme’s low affinity for pyruvate (Zhang et al., 2011).

In order to push the reversible carboxylation of pyruvate for efficient L-malic acid production, an NADP^+^-dependent malic enzyme from *Arabidopsis thaliana* (NADP-ME_2_) with higher affinity for pyruvate was selected and modified by site-directed mutagenesis. The best mutant ME_2_(C490S) showed a 56% improvement of malate synthesis activity, and its introduction into *E. coli* F0501 (Δ*ldhA*, Δ*poxB*, Δ*pflB*, Δ*pta* and Δ*ackA*), decreased pyruvate accumulation and the titer of L-malate increased by 83%. Further inactivation of succinate synthesis genes enhanced the malic acid titer to 7.78 g/L and overexpression of *S. cerevisiae* NADH kinase (Pos5), which phosphorylates NADH to generate NADPH, resulted in the maximal titer of 21.65 g/L with a yield of in a 0.48 mol/mol in a 5 L bioreactor fermentation. However, 16.54 g/L pyruvate was still accumulated as the main by-product. This study demonstrated the potential utility of the one-step pathway for efficient L-malic acid production, whereby elevating NADPH levels is a key factor (Dong et al., 2017). More recently, a recombinant expression strategy was employed to co-localize the native pyruvate kinase (PykF) and malic enzyme (SfcA) by forming an enzyme scaffold complex in *E. coli*. The close co-localization of PykF and SfcA can increase the pathway flux from pyruvate to malic acid in the one-step pathway. In a flask culture, the recombinant strain harboring the scaffold complex produced a maximal malic acid titer of 5.72 g/L from 10 g/L glucose, which was significantly increased 2.1-fold. In a 5-L bioreactor, the malic acid production reached 12.08 g/L (Somasundaram et al., 2018).

### Malic Acid Export

An important aspect of organic acid production in microbial cell factories is the export of the product across the cell membrane into the culture supernatant (Sauer et al., 2008). Export of products can not only reduce the toxicity of intracellular product accumulation, but also increase the product yield by relieving negative feedback regulation. As stated above, enhancing the capacity of L-malic acid excretion by genetic modification is a highly effective strategy that has been widely employed in filamentous fungi and yeasts (Zelle et al., 2008; Brown et al., 2013; Zambanini et al., 2017; Li et al., 2019; Xu et al., 2019). The Mae1 malic acid transporter from *S. pombe* was overexpressed in several fungi to improve their malic acid production as mentioned above (Zelle et al., 2008; Chen et al., 2013; Liu et al., 2017a; Chen et al., 2017). Notably, its expression in *S. cerevisiae* increaseed malic acid production threefold (Zelle et al., 2008). Additionally, the SpMae1 homologs C4t318 and AcDct were identified in *A. oryzae* and *A. carbonarius*, respectively (Brown et al., 2013; Yang et al., 2017a). Although the mechanism of these malic acid export proteins has been partly elucidated, there is still limited information on the control of the metabolic flux towards malic acid through manipulation of malic acid transporters in microbial cell factories. Originally, SpMae1 was annotated as a member of the TDT family and was believed to use the proton motive force to transport malate, succinate and malonate in *S. pombe* (Grobler et al., 1995). Now it is clear that AcDct and SpMae1 belong to the voltage-gated anion channel family SLAC1 (slow-anion channel), which do not use protons, Na^+^, or ATP (Darbani et al., 2019). Such transporters offer a tremendous advantage for fermentative organic acid production, allowing a higher overall product yield due to their lower energy requirements. More recently, we tested the impact of deleting five putative C4-dicarboxylate transporters (*dct1*, *dct2*, *dct3*, *dct4* and *dct5*) in *A. niger*, and found that Dct1 (ASPNIDRAFT_193,984) was the key malic acid exporter. Deletion of Dct1 resulted in almost complete absence of malic acid accumulation, while its overexpression significantly improved the malic acid yield (Cao et al., 2020).

Transporter engineering to improve the malic acid production of bacteria is rarely reported. The three transporters DcuA, CitT, and TtdT were identified as the major malic acid export proteins of *E. coli* (Kurgan et al., 2019). Inactivation of each one significantly decreased the malic acid titer, but the overexpression of each one resulted in no improvement of malic acid production (Trichez et al., 2018; Kurgan et al., 2019). This suggests that any of the malic acid transporters is sufficient for current production metrics, or there is a limitation of counter ions for the current transport mechanism. Moreover, transporter redundancy is a common phenomenon in organic acids export in *E. coli*. For instance, cells were still capable of producing 30% of the final malic acid titer of the reference strain XZ658 after the simultaneous deletion of *dcuA*, *citT*, and *ttdT* (Kurgan et al., 2019).

### Elimination of By-Product Accumulation

Microbial production of malic acid is normally accompanied by the accumulation of high levels of various by-products, mainly including other organic acids (Table 3). For instance, *E. coli* KJ071 and W3110 respectively accumulated 33.07 g/L succinate and 16.54 g/L pyruvate (Jantama et al., 2008; Dong et al., 2017), while *S. cerevisiae* produced more than 30 g/L pyruvate in the process for malic acid fermentation (Zelle et al., 2008). The concentration of these by-products was more than half the concentration of malic acid. Similarly, despite the high titers of malic acid achieved using filamentous fungi such as *A. oryzae* and *M. thermophila*, they also produced around 19 g/L succinate at the same time (Liu et al., 2017a; Li et al., 2019). The biosynthesis of these by-products not only consumes carbon sources, thereby reducing the yield of the target product, but also increases the cost of downstream product separation and purification. As a consequence, inhibition of by-product formation in various fermentation processes has also been the focus of research.

*E. coli* cannot accumulate malic acid under normal circumstances (Martinez et al., 2018), but it produces high levels of acetate, lactate, ethanol and formate during glucose fermentation. Blocking the synthesis of these by-products is vital for engineering *E. coli* to accumulate high levels of malic acid. Thus, genetic modification of *E. coli* usually starts by deleting genes encoding key enzymes of competing pathways (Jantama et al., 2008; Zhang et al., 2011; Guo et al., 2018). Following the deletion of *ldhA*, *adhE*, *ackA*, *focA*, *pflB* and *mgsA* in the evolved strain *E. coli* C, the highest malic acid titer of the resulting strain KJ071 reached 69.14 g/L. However, the strain also accumulated 33.07 g/L succinate (Jantama et al., 2008). The succinate-producing *E. coli* KJ073 (Δ*ldhA*, Δ*adhE*, Δ*ackA*, Δ*focA*, Δ*pflB*, Δ*mgsA* and Δ*poxB*) was also modified to produce malic acid (Zhang et al., 2011). Inactivation of fumarate reductase (Δ*frdBC*) eliminated over 90% of succinate production, but also led to an increase of pyruvate and decrease of acetate. After deletion of malic enzyme genes (*scfA* and *maeB*), pyruvate production was almost completely eliminated and malic acid production was further increased. Additional deletion of the three fumarase genes (*fumABC*) increased the malic acid titer 4-fold but caused a large and unexpected increase of lactate production. The final strain XZ658 produced 34 g/L malic acid with a yield of 1.42 mol/mol glucose, achieving an over 500-fold increase of the malate titer using a two-stage process (aerobic cell growth and anaerobic malic acid production). At the same time, the by-product titers of succinic, lactic and acetic acid were dramatically decreased to 1.18, 1.08 and 0.48 g/L, respectively (Zhang et al., 2011). In *E. coli*, all these by-products are derived from pyruvate, and inhibition of pyruvate formation is an effective strategy for reducing by-product biosynthesis and PEP consumption (Zhu and Tang, 2017). For instance, deletion of pyruvate kinase (*pykA* or *pykF*) reduced lactate production by over 90% (Zhang et al., 2011). However, pyruvate is also an important intermediate in the synthesis pathway of malic acid (Zelle et al., 2010; Dong et al., 2017), and disruption of the key genes in the relevant pathways would also reduce the malic acid production. Thus, blocking pyruvate secretion may be more effective in this situation. Conceptually similar strategies were proven successful in the metabolic engineering of host strains to produce L-arginine and 5-aminovalerate (Park et al., 2014; Li Z. et al., 2016).

Two strategies were developed to reduce succinate accumulation in *A. oryzae* (Liu et al., 2018). The first strategy is based on the fact that the intracellular succinate and fumarate are mainly present in the cytosol and mitochondria. The dicarboxylate carrier Sfc1p from *S*. *cerevisiae* is an antiporter that imports succinate into mitochondria and exports fumarate into the cytosol. When it was overexpressed, the succinate by-product titer was decreased, while malic acid production was increased due to more fumarate being converted into malic acid in the cytosol. This strategy might also be suitable for the metabolic engineering of other fungi or yeasts to weaken the accumulation of succinate. Given that excess supply of NADH may accelerate succinate synthesis, the second strategy is based on tuning the intracellular redox potential to reduce the NADH/NAD^+^ ratio by overexpressing the NADH oxidase (NOX) from *Streptococcus lactis*. To the end, L-malate titer of the engineered *A. oryzae* strain finally increased to 117.2 g/L and the by-product succinate titer decreased to 3.8 g/L. However, a very low level of NADH was unfavorable for malic acid synthesis (Liu et al., 2018).

Oxalate and citrate are the main by-products accompanying malic acid production in *A*. *niger* as mentioned above (Xu et al., 2019). Oxalate can be completely eliminated by deletion of *oahA*, but the resulting strain still accumulated 28.00 g/L of citric acid in fed-batch fermentation (Xu et al., 2019). Hence, the accumulation of citrate is one of the main unaddressed issues in the application of *A. niger* for malic acid production. In order to eliminate or reduce the synthesis of citrate, we recently explored the effects of two different potential targets, the global regulator LaeA and the citric acid transporter CexA, which respectively affect citric acid production and transport (Niu et al., 2015; Steiger et al., 2019), in the malic acid-producing strain *A*. *niger* S575. It was found that disruption of *cexA* could abolish the accumulation of citric acid (Xu et al., 2020). This strategy might also be suitable for the metabolic engineering other fungi such as *A. carbonarius* to eliminate the accumulation of citrate. The strategy of deleting by-product exporters to block their extracellular accumulation may not be applicable to other C_4_-dicarboxylic acids such as succinate and fumarate. Firstly, no specific fumarate or succinate transporter was identified to date. Moreover, the known C_4_-dicarboxylate transporters such as SpMae1, AcDct and DctA are generally responsible for the export of several C_4_-dicarboxylic acids, including malic acid (Janausch et al., 2002; Valentini et al., 2011; Yang et al., 2017a; Darbani et al., 2019). Hence, enhancing these exporters is necessary for improving malic acid production. Additionally, agitation rate, nitrogen, Fe (Ⅱ) ion and phosphate concentrations were also found to be impact factors of L-malate and othe C4-dicarboxylate accumulations in a 16-L stirred fermentor by *Aspergillus flavus* (Battat E. et al., 1991).

In brief, there are three potential strategies of eliminations of the byproducts: 1) deleting or weakening competing pathways for biosynthesis or transport process, 2) mining more efficient key enzymes at the metabolic node for L-malate biosynthesis, 3) optimizing fermentation process parameters.

### Enhancing Metabolic Fluxes to Improve the Production of L-Malic Acid

Phosphofructokinase-1 (Pfk1), which catalyzes the irreversible ATP-dependent phosphorylation of D-fructose 6-phosphate to fructose 1,6-bisphosphate, is the rate-limiting enzyme of the glycolytic pathway. Its activity is affected by a series of intracellular compounds such as citrate, ATP, cAMP, ammonia or trace metals (Mn and Mg) (Habison et al., 1983; Arts et al., 1987). Previously, it was considered to be a major regulatory enzyme for metabolic flux control during the production of citric acid in *A. niger* (Yang et al., 2017b). Overexpression of a truncated Pfk1, which is not inhibited by intracellular citrate, resulted in enhanced citric acid production in *A. niger* (Ruijter et al., 1997; Capuder et al., 2009). Increasing the metabolic flux in the glycolytic pathway to improved citric acid production provided a good basis for the production of malic acid when combined with other genetic modifications in other strains. Guided by transcription analysis of the expression profiles of key genes related to L-malic acid synthesis, Pfk was identified as a potential rate-limiting enzyme for L-malate production in a malic acid-producing strain of *A. oryzae*. Overexpression of *pfk* under the control of the strong and inducible *sodM* promoter twsited the repression of *pfk* expression probablely caused by malate accumulation. Based on the above changes, the L-malate titer increased from 89.5 to 93.2 g/L in shake flasks (Liu et al., 2017a).

The engineered malic acid-producing strain *M. thermophila* JG207 is distinct from *A. oryzae* because it does not show changes in the transcription levels of the glycolytic pathway genes compared with the wild type when grown on glucose, in spite of more efficient substrate utilization. However, strain JG207 showed markedly higher expression levels of multiple sugar transporter genes with either glucose or cellulose as substrate. Further enhancing glucose transportation by heterologous expression of the low-affinity glucose transporter GLT-1 from *Neurospora crassa* efficiently improved the conversion of substrates, and also increased malic acid production (Li et al., 2019).

In addition to Pfk, there are two other irreversible steps in the glycolytic pathway, phosphorylation of glucose catalyzed by hexokinase (Hxk)/glucokinase (Gk) and phosphate transfer from phosphoenolpyruvate to ADP during the production of pyruvate by pyruvate kinase (Pki), may also play a role in the regulation of the metabolic flux in glycolysis (Yang et al., 2017b). Recently, we tested Hxk, Pfk and Pki, as well as the glucose transporter MstC in the *cexA*-disruption strain mentioned above. Individual overexpression of these genes increased malic acid accumulation, and the co-overexpression of these four genes significantly improved the malic acid yield on glucose from 1.27 to 1.64 mol/mol (Xu et al., 2020).

## Conclusions and Perspectives

Metabolic engineering has been developed into a powerful tool for understanding the mechanism of malic acid biosynthesis, and also greatly promoted the progresses of engineering in *E. coli*, yeasts, and filamentous fungi for malic acid production. Since bacteria such as *E. coli* are generally not good natural malic acid production strains and also accumulate many by-products such as acetate, lactate, ethanol and formate, introduction of heterologous genes or pathways to reconstruct biosynthesis pathways with combinational deletion of genes from competing pathways is the most common strategy. While some yeasts and filamentous fungi can naturally produce high amounts of malic acid, combined enhancement of their native synthetic pathways, generally the rTCA pathway, with increased export of malic acid from the cell could dramatically improve the product titer. Efficient strategies for eliminating by-products such as succinic and citric acid, as well as the enhancement of relevant metabolic fluxes have also been exploited to increase the malic acid yield in *A. niger*, *A. oryzae* and *M. thermophila* (Li et al., 2019; Xu et al., 2019; Liu et al., 2018). Compared with *S. cerevisiae* and prokaryotes, the malic acid titers of filamentous fungi were usually higher (Table 1). Therefore, filamentous fungi are considered the most promising host strains for the microbial fermentation of malic acid. Notably, the engineered *A. niger* S575 with GRAS status produced the highest malic acid titer reported to date, and after elimination of the by-product citric acid and enhancement of the main metabolic flux, the yield of malic acid from glucose was further improved. The elimination of major by-products can significantly decrease the cost of downstream processing by simplifying separation and purification. Additionally, 50% of the total cost is used for the separation and extraction process of malic acid produced by microbal fermentation (Dai Z. et al., 2018). Nevertheless, the industrial success of biosynthesis is ultimately based on rapid and economical conversion of substrates into target products, so from the view of industrialization, the next reconstructive emphasis will concern on: 1) shortening the fermentation period, 2) identifying the limiting factors for the efficient use of cheap carbon feedstocks, 3) exploring the regulatory factors of L-malate synthesis pathway to improve production efficiency, 4) reducing by-product synthesis to increase L-malate yield and reduce the costs of downstream separation and extraction.

Low-cost sugar feedstocks are preferred for large scale fermentation for increase of profit margin. The price of raw materials accounts for a large proportion of the total production cost in industrial fermentation processes. However, the substrate used for microbial fermentation of malic acid is mostly the relatively expensive glucose. Accordingly, the selection of more economical renewable feedstocks for malic acid production, such as lignocellulosic biomass from agricultural waste or crude glycerol from the biodiesel industry, has received increasing attention. Biotechnological processes have shown great potential to utilize these cheap feedstocks for malic acid production (Zambanini et al., 2016b; Li et al., 2019). Metabolically engineering the most promising strains to develop versatile processes which can be adapted to cost-effective feedstocks may be another important subject of future research.

In all the current processes of microbial fermentation for malic acid production, large amounts of CaCO_3_ must be added as a neutralizing agent to keep the culture pH constant at around 6.5. As a consequence, the fermentation end-product is calcium malate formed in the bioreactor, which requires cost-intensive acidification and precipitation for conversion into pure malic acid during downstream processing. Systems biology or the latest genome-scale metabolic models can provide solutions to complex metabolic engineering goals of industrial importance (Upton et al., 2020), and further genetic engineering of malic acid-producing strains of *Aspergillus*, which have extremely high natural acid tolerance, to produce malic acid at low pH values would be a promising approach to avoid the excessive addition of neutralizing agents.
